# Increasing complexity in children with life-limiting conditions in England: an analysis of national routine data

**DOI:** 10.1136/archdischild-2026-330504

**Published:** 2026-07-13

**Authors:** Bethan Page, Stuart William Jarvis, Lorna K Fraser

**Affiliations:** 1 King’s College London, London, UK; 2 Health Sciences, University of York, York, UK; 3 Cicely Saunders Institute, King’s College London, London, UK

**Keywords:** Epidemiology, Palliative Care, Paediatrics, Health Policy, Child Health Services

## Abstract

**Introduction:**

The number of children with life-limiting conditions has risen in recent decades, partly due to medical advances enabling increased survival. It is unclear how the medical complexity of this population has changed.

**Aim:**

To use quantitative analyses of routinely collected healthcare and administrative data from 2003 to 2022 to assess the complexity of children with a life-limiting condition (0–19 years) over time.

**Methods:**

National hospital data in England (Hospital Episode Statistics) were used to identify children aged 0–19 years with a life-limiting condition from 2003/2004 to 2022/2023. Data were linked with community pharmacy data. Indicators of medical complexity were assessed based on the domains of the medical complexity framework: number of life-limiting and chronic conditions, number of main specialties involved, number of medications, healthcare use and technology dependence (gastrostomies, tracheostomies, jejunostomies) with trends summarised descriptively and associations assessed using multivariable ordinal logistic regression.

**Results:**

Most indicators of complexity increased over time, including numbers of health conditions, specialties, technology dependence, emergency department attendances and emergency admissions. Technology dependence rose, particularly for gastrostomies (3505 to 15 949). Children seen by three or more specialties increased from 3096 to 19 312. Rising healthcare use reflected both increasing numbers of children with life-limiting conditions and increasing complexity among individual patients. Increasing age, increasing deprivation and Bangladeshi and Pakistani ethnicity were associated with greater complexity.

**Conclusions:**

Healthcare providers must allocate resources to address the increasing complexity of need in children with life-limiting conditions. No data were available on family impacts or family-identified needs.

**WHAT IS ALREADY KNOWN ON THIS TOPIC**

- The prevalence of children with life-limiting and life-threatening conditions has increased in recent decades.
- There is a higher prevalence in areas of higher deprivation and in some ethnic minority groups.
- Clinicians report that children with life-limiting conditions present with increasingly complex health needs.

**WHAT THIS STUDY ADDS**

- There have been substantial rises in many indicators of complexity in children with life-limiting conditions over the past 19 years in England.
- Older age, higher levels of deprivation and Bangladeshi and Pakistani ethnicity are all associated with greater levels of complexity.

**HOW THIS STUDY MIGHT AFFECT RESEARCH, PRACTICE OR POLICY**

- Future research should address family and social aspects of complexity.

## Introduction

 The number of children living with life-limiting conditions has increased over the past two decades in the UK^1 2^ and other high-income countries,^3–6^ mainly due to increasing survival.^2 7^ Previous research found that the absolute number of children with a life-limiting condition in England rose from 30 535 in 2001/2002 to 86 625 in 2017/2018.^2^ Life-limiting conditions include those with no reasonable hope of cure and life-threatening conditions for which curative treatment may be feasible but can fail, for example, cancer.^8^ Together, these conditions (hereafter referred to as life-limiting conditions) represent a heterogeneous group with nearly 400 diagnoses.^9^ The term complex chronic conditions is used to describe a similar population.^6^

Clinicians report increasing complexity in children with life-limiting conditions, but data quantifying complexity over time are lacking.^10^ Studies have shown an increase in some domains of complexity such as the use of medical technologies like home ventilation.^11 12 12^ A US study reported that the proportion of hospital resource use attributable to children with complex chronic conditions had increased from 2000 to 2022.^13^ Understanding how the complexity of children with life-limiting conditions is changing is key for policymakers and service providers to understand how best to adapt and plan services.

Previous studies have operationalised a measure of complexity based on a widely recognised definition of medical complexity with four domains: chronic conditions, healthcare usage, functional limitations and family needs.^14^ No study has yet examined long-term population-wide trends in complexity in England.

This study uses analyses of routinely collected healthcare and administrative data from 2003/2004 to 2022/2023 to assess the complexity of the population of children with a life-limiting condition (0–19 years) in England over time.

## Methods

### Data sources

Individual-level pseudonymised patient data were obtained from NHS England for all children and young people aged 0–19 years at any point between 1 April 2003 and 31 March 2023 with a life-limiting condition present in the Admitted Patient Care (APC) data. The datasets listed below were linked by a unique patient pseudo-ID.

- Hospital Episode Statistics Admitted Patient Care (HES APC): identification of life-limiting conditions, presence in England with inpatient admission, demographic data, healthcare use (admissions and emergency admissions) and complexity (emergency admissions and length of stay).
- Hospital Episode Statistics Accident and Emergency (HES A&E) (2007–2019): A&E visits.
- Emergency Care Data Set (2019–2023): A&E visits.
- Medicines Dispensed in Primary Care (NHS Business Services Authority data) (2018–2023): community prescriptions.

A repeated cross-sectional analysis was used to assess period prevalence each year using denominator population data from Ethnic group population trends and projections for UK local areas

(http://ethpop.org).

### Study population

Children were included if they had a record of any of the life-limiting or life-threatening conditions recorded in their hospital admission data while aged 0–19 years. The study uses an established diagnostic coding framework using 777 four-digit International Classification of Diseases, 10th Revision (ICD-10) codes (online supplemental table 1).^9^

Eligibility criteria:

1. Had a diagnosis of one of the life-limiting condition ICD-10 codes in the current year or a previous year (from April 2003) or were seen by a paediatric palliative medicine consultant.
2. Had a hospital admission in the year of analysis (of any length, including day cases).
3. Were ≤19 years old.
4. Were resident in England.

A child will appear in the data in multiple years providing they meet the eligibility criteria for that year, that is, they need to have had a hospital admission of any length, including day cases that year.

### Demographic data

Age was set to age at the first hospital inpatient admission in the year. Sex was set to the most commonly recorded from all available records in the hospital inpatient data (HES APC).

Ethnic group data were assigned from hospital inpatient data recorded using census 2001 categories^15^: Bangladeshi, black, Chinese, Indian, mixed, other ethnic group, Pakistani and white. The most commonly recorded of these categories was assigned, excluding other ethnic group or missing. Other ethnic group was only assigned if there was no other ethnicity recorded in the patients’ records: the number of patients with ‘Other Ethnicity’ had increased markedly in recent years likely due to inaccuracies in recording of ethnicity.

An Index of Multiple Deprivation 2010 score was assigned to each individual based on the 2001 Lower-layer Super Output Area of residence in that year, and five population-weighted categories were created (from 20% least deprived to 20% most deprived).

### Complexity

Complexity scoring is built on Cohen’s conceptualisation of medical complexity^14^ and previous attempts to operationalise this.^16 17^ Trends in individual components were described over time. Data for components using HES APC data only were available from 2003/2004. For the more recent years, a composite complexity score was developed by summing over five domains of complexity, with each domain given a score of 0 or 1 (figure 1), giving an overall score from 0 to 5 for each individual in each year. Composite complexity could be assessed from 2018/2019 onwards due to data availability.

**Figure 1:**
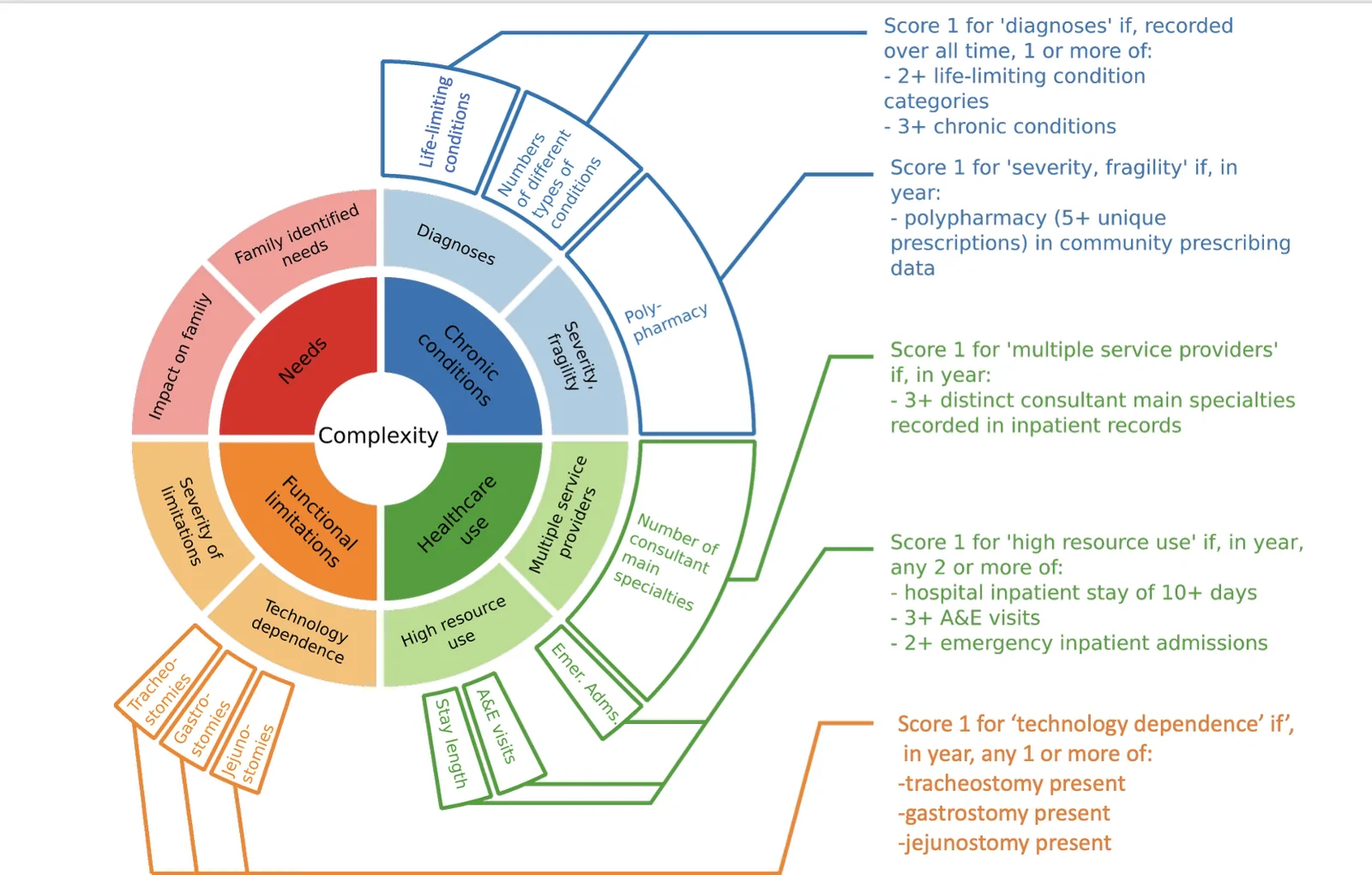
Conceptualisation and scoring system for medical complexity. Complexity is split across four main domains and eight subdomains, five of which can be assessed from items recorded in routine records as indicated. A&E, accident and emergency; Emer Adms, emergency admissions.

- Diagnoses: life-limiting conditions were assessed using categories described in online supplemental table 1. Chronic conditions were assessed by matching a previously developed list of chronic conditions^18^ to inpatient diagnoses (HES APC).
- Polypharmacy: five or more unique prescriptions are a previously used definition of polypharmacy.^19^ PAIDBNF code was used to identify medications (a 15-digit identifier used to uniquely identify a medicine, its formulation and strength for reimbursement purposes).^20^ Medications with the same codes for the first five levels of the British National Formulary code were considered the same (ie, chapter, section, paragraph, subparagraph and chemical substance).^21^
- Multiple service providers: unique consultants’ main specialties in each year were counted from inpatient data using the MAINSPEF (main specialty) and TRETSPEF (treating specialty) fields (HES APC).
- High resource use: hospital events were counted including total A&E visits, inpatient admissions, emergency admissions and inpatient bed days for each person in each year. Length of stay was also assessed per year.
- Technology dependence: for gastrostomies, tracheostomies and jejunostomies, relevant ICD-10 and **Office of Population Censuses and Surveys Classification of Interventions and Procedures** (OPCS-4) were searched for in inpatient (HES APC) records. Medical technologies were assumed to still be in situ unless a code indicating their removal was recorded.

### Data management

Data were managed in PostgreSQL. Analyses and graphs were produced using RStudio V.4.42 and Excel.

### Analyses

The numbers of children (0–19 years) with a life-limiting condition and the prevalence of life-limiting conditions within the population of 0–19 year-olds in England were calculated. The prevalence and 95% CIs were calculated per 10 000 populations at risk by dividing the number of individuals with a life-limiting condition by the population estimates for children aged 0–19 years in England that year based on the ETHPOP dataset. Prevalence was calculated as an overall total per 10 000 and stratified by age group, ethnicity and deprivation category. Complexity was described by key demographic and clinical variables. A multivariable ordinal logistic regression model was run to assess the association between composite complexity score and key demographic variables for 2022/2023. Given the large sample size, emphasis was placed on the magnitude and clinical relevance of observed changes rather than statistical significance alone.

## Results

### Prevalence

The absolute number of children (0–19 years) with a life-limiting condition increased from 31 298 in 2003/2004 to 87 885 in 2018/2019 and has plateaued since—88 722 in 2022/2023 (figure 2, online supplemental table 2). The prevalence increased from 25.3 per 10 000 children aged 0–19 years in England (95% CI 25.0 to 25.6) in 2003/2004 to 64.2 per 10 000 in 2017/2018 (95% CI 63.8 to 64.7) and has steadied since then—65.0 per 10 000 in 2022/2023 (95% CI 64.6 to 65.4). The numbers in 2020/2021 (during the COVID-19 pandemic) were lower (67 400; 50.4 per 10 000, 95% CI 50.1 to 50.8).

**Figure 2:**
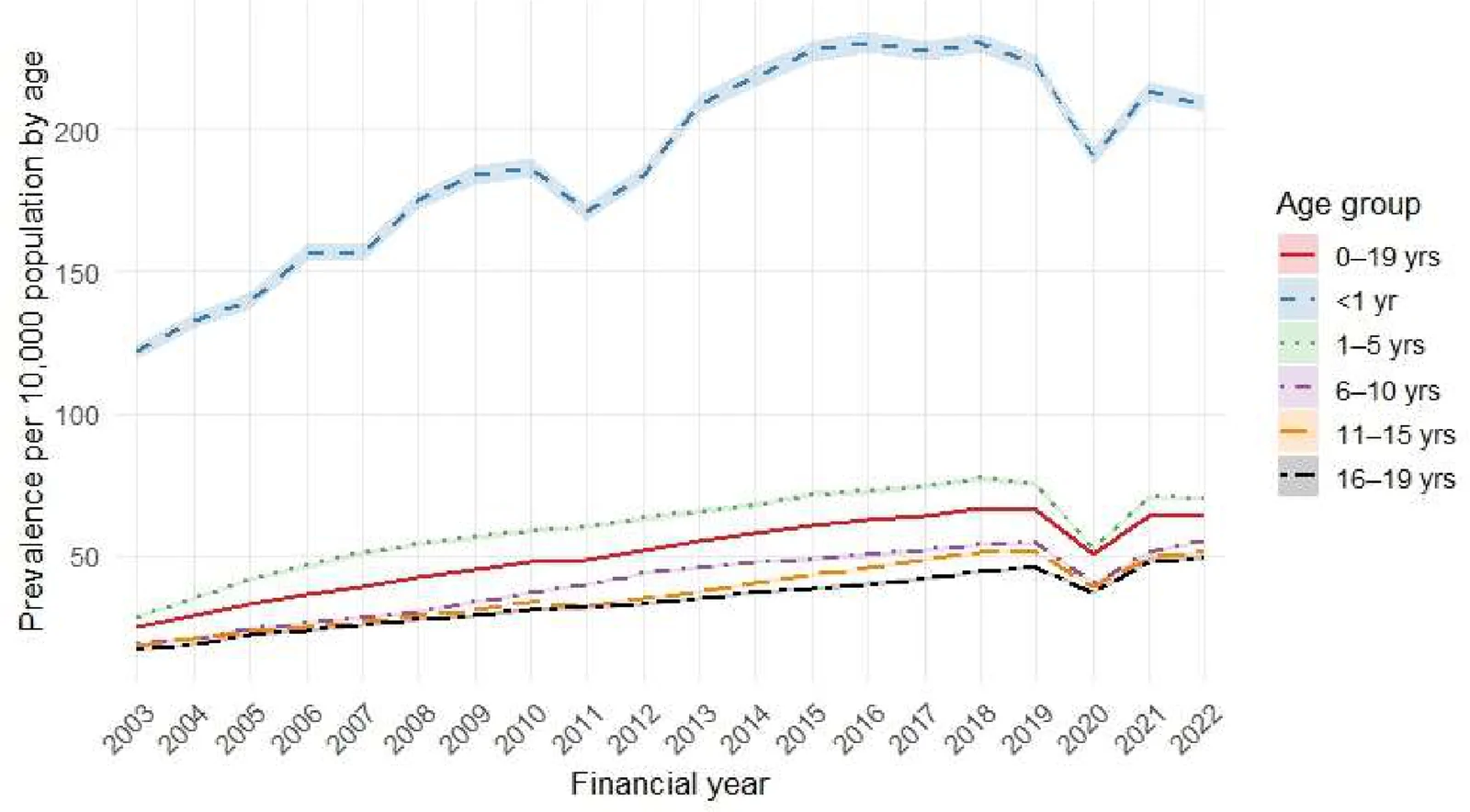
Prevalence per 10 000 populations of life-limiting conditions among children aged 0–19 years in England, 2003–2022 by age group.

Prevalence was highest in under 1s (figure 2, online supplemental table 2). The largest proportional increases were seen in older age groups. There was some evidence that the prevalence has reduced slightly in under 5s since 2018/2019.

There was a higher prevalence in males than females: 70.9 per 10 000 (95% CI 70.3 to 71.5) in males and 58.5 (95% CI 58.0 to 59.1) in females in 2022/2023. Prevalence was higher in many ethnic minority populations, highest in the Pakistani population (99.1 per 10 000, 95% CI 96.6 to 101.5) (online supplemental figure 1).

Children with life-limiting conditions were more likely to live in more deprived areas: in 2022/2023, 26% were in the most deprived group and 15% in the least deprived group, despite each group representing 20% of that age group in the general population (online supplemental figure 2).

The highest prevalence was for congenital diagnoses at 27.6 per 10 000 (95% CI 27.3 to 27.8) in 2022/2023 (online supplemental table 3). There was an increase in prevalence in all diagnostic groups from 2003/2004 to 2022/2023, with the greatest increases for perinatal, gastrointestinal and other diagnostic groups. Oncology diagnoses had the lowest proportional growth.

### Complexity of the population

Data for each domain of complexity are summarised in table 1.

**Table 1:** Indicators of complexity over time (2003/2004–2022/2023), showing the number (n) and the percentage (%) of children aged 0–19 years with life-limiting conditions meeting each criterion

	2003/2004	2012/2013	2022/2023
	n (%)	n (%)	n (%)
Chronic conditions			
2+ life-limiting conditions	4222 (13)	17 374 (26)	26 908 (30)
3+ chronic conditions	10 029 (32)	42 088 (63)	60 112 (68)
Polypharmacy (5+ unique prescriptions)	N/A	N/A	40 446 (46)
Healthcare usage			
3+ main specialties	3096 (10)	15 792 (24)	19 312 (22)
Inpatient stay of 10+ days	8588 (27)	11 829 (18)	12 161 (14)
3+ ED visits	N/A	7718 (12)	13 528 (15)
2+ emergency admissions	8795 (28)	16 936 (25)	21 515 (24)
Functional limitations			
Gastrostomy	3505 (11.2)	11 859 (19.9)	15 949 (20.1)
Tracheostomy	633 (2.0)	2010 (3.0)	2276 (2.6)
Jejunostomy	77 (0.2)	798 (1.2)	1079 (1.2)

ED, emergency department.

#### Chronic condition domain

There was a sixfold increase in the number of children with two or more life-limiting conditions over the study time period (2003/2004: 4222; 2022/2023: 26 908) (online supplemental figure 3). The proportion of the cohort with 2+ life-limiting conditions increased from 13% in 2003/2004 to 30% in 2022/2023. There were 8236 children with 3+ life-limiting conditions in 2022/2023 (9% of the cohort), up from 421 in 2003/2004 (1% of the cohort).

There was a sixfold increase in the number of children with 3+ chronic conditions (2003/2004: 10 029; 2022/2023: 60 112) (online supplemental figure 4). In 2003/2004, 32% had 3+ chronic conditions, increasing to 68% in 2022/2023.

Medication data were available from 2018/2019 to 2022/2023. The absolute number and proportion of the cohort with polypharmacy has increased slightly (2018/2019: 36 096 (41%); 2022/2023: 40 446 (46%)) (online supplemental figure 5).

#### Healthcare use domain

The number of children cared for by three or more inpatient specialties increased sixfold between 2003/2004 and 2016/2017 and has plateaued since (2003/2004: 3096; 2016/2017: 19 664; 2022/2023: 19 312) (online supplemental figure 6). The proportion seen by 3+ specialties increased (2003/2004: 10%; 2022/2023: 22%), and the proportion seen by only one specialty decreased (2003/2004: 63%; 2022/2023: 42%).

Emergency department (ED) data were available from 2007/2008 to 2022/2023. The number of children with 3+ ED attendances increased fourfold (2007/2008: 3598; 2022/2023: 13 528) (online supplemental figure 7). The proportion with 3+ ED visits increased (2007/2008: 7%; 2022/2023: 15%), and the proportion with no ED visits decreased (2007/2008: 69%; 2022/2023: 52%).

The absolute number of children with 2+ emergency inpatient admissions increased fourfold (2003/2004: 8795; 2022/2023: 21 515), and the cohort proportion decreased slightly (2003/2004: 28%; 2022/2023: 24%) (online supplemental figure 8).

The number of day cases increased (2003/2004: 78 248; 2022/2023: 196 016) (online supplemental figure 9). The number with at least one hospital admission of 10 or more days increased slightly from 2003/2004 to 2012/2013 and has remained stable since (2003/2004: 8588; 2012/2013: 11 829; 2022/2023: 12 161) (online supplemental figure 10). The proportion of the cohort with an inpatient admission of 10+ days per year decreased (2003/2004: 27%; 2022/2023: 14%).

#### Functional limitations domain

The number of children with a gastrostomy increased from 3505 (11.2%) in 2003/2004 to 15 949 (20.1%) in 2022/2023 (online supplemental figure 11). Those with a tracheostomy rose from 633 (2.0%) to 2276 (2.6%), and those with a jejunostomy increased from 77 (0.2%) to 1079 (1.2%) during the same time period. The number of children with medical technologies has plateaued since 2018/2019.

### Composite complexity scores

A composite complexity score for children with a life-limiting condition was able to be calculated for the most recent 5 years’ worth of data. This showed a range of values with all possible scores present, although scores of 0, 1 or 2 were most common with 22–31% in each (figure 3, online supplemental table 4). The presence of complexity across all five domains was rare (1%), but 6% of the group could be considered highly complex (scores of 4 or 5). As data for all domains of complexity were only available since 2018, it is difficult to assess change in composite complexity score over time.

**Figure 3:**
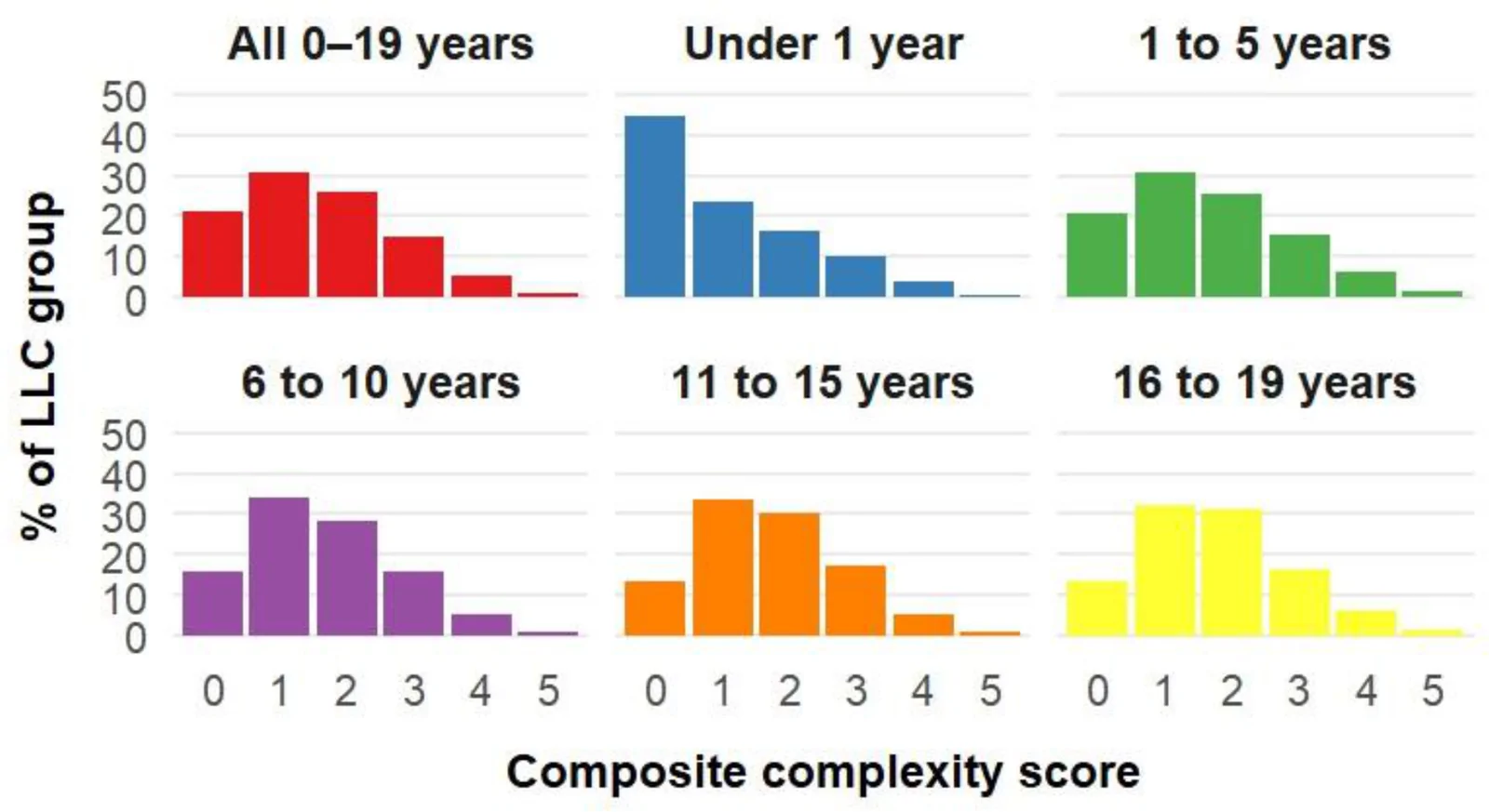
Composite complexity score by age group, 2022–2023 financial year. Percentage of the population of children with a life-limiting condition (LLC) for each complexity score from 0 to 5 presented by age group.

Lower scores for complexity were much more common in children under 1 year than in other age groups, with 68.3% scoring 0 or 1, whereas <50% scored 0 or 1 in children over 6 years. In under 1s, only 4.8% scored 4 or 5, whereas in 16–19 year-olds, 7.8% scored 4 or 5 (figure 3, online supplemental table 4).

The proportion of those with scores of 4 or 5 (highly complex) was highest for other diagnoses (26%), neurology (15%), genitourinary (14%) and respiratory (13%), as well as lowest for perinatal (6%), congenital (7%) and oncology (7%) (online supplemental figure 12). The distribution is markedly different for perinatal, with 0 as the most common composite complexity score.

Composite complexity score varied by ethnic group, with some ethnic groups like Pakistani and Bangladeshi having higher composite complexity scores compared with white British/Irish (online supplemental figure 13). There was a slight trend towards increasing complexity in the most deprived groups (online supplemental figure 14).

The multivariable ordered logistic regression model assessing associations between composite complexity score and key demographic variables showed that being from Pakistani ethnicity (OR=1.52, 95% CI 1.45 to 1.59) or Bangladeshi ethnicity (OR=1.45, 95% CI 1.32 to 1.59) was associated with higher complexity scores when compared with white British children (table 2). Increasing age was associated with increasing complexity (OR=1.05 per year of age, 95% CI 1.05 to 1.05). Higher deprivation was associated with increasing complexity (OR=1.24, 95% CI 1.20 to 1.29 for the 20% most deprived compared with the 20% least deprived). Missing data for deprivation were associated with higher complexity scores and missing data for ethnicity were associated with lower complexity scores.

**Table 2:** Multivariable ordered logistic regression model predicting composite complexity score for 2022–2023 data, adjusted for age, ethnicity and deprivation

	n (%)	OR (95% CI)	P value
Ethnic group			
White	62 433 (70.4)	1 (ref)	
Bangladeshi	1539 (1.7)	1.45 (1.32 to 1.59)	<0.001
Black	5412 (6.1)	1.04 (0.98 to 1.09)	0.176
Chinese	291 (0.3)	0.88 (0.72 to 1.09)	0.241
Indian	2609 (2.9)	1.07 (0.99 to 1.15)	0.063
Mixed	4346 (4.9)	0.93 (0.88 to 0.98)	0.009
Other	4156 (4.7)	1.06 (1.01 to 1.13)	0.035
Pakistani	6260 (7.1)	1.52 (1.45 to 1.59)	<0.001
Missing/unknown	1676 (1.9)	0.28 (0.26 to 0.31)	<0.001
Age at start of year			
Age (years)*		1.05 (1.045 to 1.049)	<0.001
Deprivation			
20% least deprived	13 505 (15.2)	1 (ref)	
20–40% least deprived	14 420 (16.3)	1.12 (1.08 to 1.17)	<0.001
40–60% middle deprived	15 888 (17.9)	1.15 (1.11 to 1.20)	<0.001
20–40% most deprived	18 922 (21.4)	1.23 (1.18 to 1.27)	<0.001
20% most deprived	24 806 (28.0)	1.24 (1.20 to 1.29)	<0.001
Missing/unknown	1111 (1.3)	1.88 (1.57 to 2.26)	<0.001

Higher ORs are associated with higher composite complexity scores.

* OR represents the change in odds per 1-year increase in age. There were no missing data for age.

## Discussion

The population of children with life-limiting conditions in England has increased over time and plateaued since 2018. The complexity of this population has increased over the past two decades, particularly for technology dependence, numbers of chronic conditions and specialties involved. Polypharmacy is evident in nearly half of this population. Healthcare usage has changed, with large increases in ED attendances and short hospital admissions. Increasing age, increasing deprivation and Bangladeshi and Pakistani ethnicity were associated with greater complexity.

### Population with life-limiting conditions

Large increases in numbers and prevalence were seen from 2003/2004 to 2018/2019, followed by a plateau that may reflect post-COVID-19 changes in hospital use and greater community care. This needs further exploration over the next 5–10 years. The dip in 2020/2021 likely reflects the effect of the pandemic on healthcare delivery. Evidence from the USA also suggests a possible plateau in the numbers of children with complex chronic conditions following large increases prior to 2016.^13^

### Increase in complexity

The increase in individual measures of complexity in this study aligns with clinical reports^10^ and evidence from the USA.^13^ UK studies have shown an increase in some domains of complexity in 14–19 year-olds with life-limiting conditions in England^17^ and a small shift towards increasing complexity in 0–25 year-olds in Wales.^16^

Our findings show marked increases in technology dependence particularly for feeding tubes, consistent with international evidence.^13 22^ Polypharmacy was present in 46% of children with life-limiting conditions in our study, a higher rate than previous UK estimates^19^ but similar to a study from Italy.^23^

Prior studies show a higher prevalence of children with life-limiting conditions in areas of higher deprivation and in some minority ethnic groups.^2 9 24^ Additionally, our findings show greater complexity among children living in deprived areas and among some minority ethnic groups.

### Implications for resource use

Increasing complexity has major implications for services.^25–27^ For some measures of resource use, both rising numbers and increased complexity contribute; for others, increased numbers alone explain higher usage. For example, the absolute numbers of children with ≥2 emergency admissions or hospital stays of ≥10 days increased, though the proportion of the total population of children with life-limiting conditions decreased.

These data are important for healthcare planners and policymakers in paediatric palliative care and in generalist settings such as EDs and pharmacies, who are seeing growing numbers of complex children and need adequate resources to care for them safely.

### Family needs domain of complexity

The only domain of complexity not quantified in this study was family needs. Previous research highlights impacts on parental physical and mental health^28^ and increased healthcare use when social care needs are present.^29 30^ Family-identified needs are more difficult to capture from administrative data. Linking health with social care data—such as parental employment, benefits and housing—is needed.

### Strengths and limitations

This study uses established frameworks for defining medical complexity and identifying children with life-limiting conditions using national, whole-population data over 19 years to examine trends.

These analyses are reliant on the quality of data collected. The variability in completeness and accuracy of coding of the ethnicity data is an example of the limitations of these data. Using data based on diagnoses alone raises some challenges, for example, coding practices change over time and can be affected by incentives to code for billing purposes. There is large variation in the severity and prognosis within some of these diagnoses, for example, quadriplegic cerebral palsy, and this is further compounded by the grouping of some diagnoses within ICD-10 since not all diagnoses have their own code. Therefore, we may be capturing some children who may not have a life-limiting condition. ED data collection was incomplete in the early years of this study, and the data collection changed in 2019/2020. The lack of primary care, community and hospice data limits completeness. Not all medical technologies (eg, home oxygen, long-term ventilation) were captured.

The earlier years may underestimate prevalence due to a left-edge effect. No data were available prior to 2003/2004. The current methodology requires a child to visit hospital (at least as a day case) to be included in a given year: a shift towards more care in the community may mean some children with life-limiting conditions are not included using this methodology. This may mean that these analyses underestimate prevalence. This method for assessing complexity is less useful in babies due to the cumulative nature of some measures, suggesting a need for alternative approaches in under 1s.

## Conclusions

Children with life-limiting conditions are increasingly complex, with important implications for service planning. Future research should address the currently unmeasured family and social dimensions of complexity.

## Supplementary material

10.1136/archdischild-2026-330504 online supplemental figure 1

10.1136/archdischild-2026-330504 online supplemental table 1

## Data Availability

Data may be obtained from a third party and are not publicly available.
