# Supplementary material for: Increasing complexity in children with life-limiting conditions in England: an analysis of national routine data
Source: Arch Dis Child. 2026 Jul 13;111(10):e330504. doi: 10.1136/archdischild-2026-330504 (PMC13638291; doi:10.1136/archdischild-2026-330504)
Supplement: Supplementary file 1 [file archdischild-111-10-s001.docx]

**Supplementary Figures**

Prevalence per 10,000 population

| Financial year | Pakistani | Other | Black | Bangladeshi | White | Mixed | Indian | Chinese |
| --- | --- | --- | --- | --- | --- | --- | --- | --- |
| 2012 | 97.3133 | 52.8511 | 58.4252 | 55.2243 | 50.343 | 35.872 | 52.5382 | 31.0999 |
| 2013 | 101.346 | 55.7463 | 62.8996 | 56.9217 | 53.5025 | 38.5441 | 54.2834 | 34.2481 |
| 2014 | 102.742 | 61.0952 | 66.686 | 58.4339 | 55.8855 | 40.7755 | 55.8437 | 32.9957 |
| 2015 | 104.957 | 65.4131 | 68.677 | 58.1186 | 58.4988 | 45.5459 | 54.7642 | 35.4681 |
| 2016 | 106.181 | 66.2357 | 69.9727 | 58.0976 | 60.2747 | 46.2821 | 54.6793 | 37.9975 |
| 2017 | 106.28 | 71.4351 | 71.6707 | 59.026 | 61.9657 | 47.989 | 55.6783 | 35.8993 |
| 2018 | 108.801 | 78.4998 | 73.5399 | 62.048 | 64.5602 | 50.9879 | 54.4908 | 33.1449 |
| 2019 | 104.9 | 79.011 | 72.5913 | 64.1805 | 64.0607 | 50.7995 | 53.8585 | 32.2249 |
| 2020 | 77.4221 | 62.1447 | 55.0155 | 50.1123 | 48.51 | 40.0081 | 40.3483 | 20.5529 |
| 2021 | 96.6205 | 82.2613 | 71.709 | 61.3921 | 61.2314 | 50.4256 | 49.9073 | 28.3347 |
| 2022 | 99.0773 | 86.5445 | 72.8885 | 62.6954 | 61.6688 | 52.4872 | 52.4556 | 27.8894 |

Supplementary Figure 1 Prevalence per 10,000 population for ethnic group of children with life-limiting conditions aged 0-19 years, 2012-2022.

Supplementary Figure 2 Distribution of children aged 0-19 years with life-limiting conditions across five deprivation categories, 2017-2022. The most deprived areas are over-represented.

Supplementary Figure 3 Number of life-limiting conditions (LLCs) in population aged 0-19 years, population size over time 2003-2022.

Supplementary Figure 4 Number of chronic conditions in the population of children with life-limiting conditions 0-19 years, 2003-2022

 

Supplementary Figure 5 Number of unique medications, numbers of children with life-limiting conditions over time, 0-19 years, 2018-2022

Supplementary Figure 6 Number of inpatient specialties in population of children with life-limiting conditions, 0-19 years, 2003-2022

Supplementary Figure 7 Emergency department visits in children with life-limiting conditions, 0-19 years, 2007-2022

Supplementary Figure 8 Number of emergency hospital admissions in population of children with life-limiting conditions, 0-19 years, 2003-2022

Supplementary Figure 9 Length of stay of hospital admissions in children with life-limiting conditions 0-19 years, 2003-2022.

  

Supplementary Figure 10 Number of children with life-limiting conditions (0-19 years) with at least 1 inpatient admission of 10 or more days, 2003-2022.

| Financial Year | Jejunostomy | Tracheostomy | Gastrostomy |
| --- | --- | --- | --- |
| 2003 | 77 | 633 | 3505 |
| 2004 | 144 | 832 | 4708 |
| 2005 | 198 | 997 | 5882 |
| 2006 | 241 | 1074 | 6571 |
| 2007 | 324 | 1261 | 7592 |
| 2008 | 393 | 1431 | 8384 |
| 2009 | 516 | 1647 | 9470 |
| 2010 | 626 | 1745 | 10282 |
| 2011 | 696 | 1858 | 11154 |
| 2012 | 798 | 2010 | 11859 |
| 2013 | 875 | 2109 | 12805 |
| 2014 | 972 | 2211 | 13523 |
| 2015 | 1039 | 2197 | 13959 |
| 2016 | 1078 | 2261 | 14611 |
| 2017 | 1145 | 2320 | 15075 |
| 2018 | 1237 | 2444 | 15944 |
| 2019 | 1240 | 2349 | 15930 |
| 2020 | 998 | 1721 | 12162 |
| 2021 | 1124 | 2233 | 15586 |
| 2022 | 1079 | 2276 | 15949 |

Supplementary Figure 11 Number of children with life-limiting conditions with gastrostomies, jejunostomies and tracheostomies over time 2003-2022

Supplementary Figure 12 Composite complexity score by diagnostic group, 2022-23 financial year. Note some children were recorded under more than one diagnostic category.

|  |  |  |
| --- | --- | --- |
|  |  |  |
|  |  |  |
|  |  |  |

|  |  |
| --- | --- |
|  |  |
|  |  |
|  |  |
|  |  |

Supplementary Figure 13 Composite complexity score by ethnic group, 2022-23 financial year

|  |  |
| --- | --- |
|  |  |
|  |  |

Supplementary Figure 14 Composite complexity score by deprivation, 2022-23 financial year
