# Supplementary material for: Increasing complexity in children with life-limiting conditions in England: an analysis of national routine data
Source: Arch Dis Child. 2026 Jul 13;111(10):e330504. doi: 10.1136/archdischild-2026-330504 (PMC13638291; doi:10.1136/archdischild-2026-330504)
Supplement: Supplementary file 2 [file archdischild-111-10-s002.docx]

**Supplementary Tables**

*Supplementary Table 1: ICD-10 (version 2016) diagnostic coding framework used to identify and categorise children with life-limiting conditions*

| Neurology | Haematology | Oncology | Metabolic | Respiratory | Circulatory | Gastrointestinal | Genitourinary | Perinatal | Congenital | | Other |
| --- | --- | --- | --- | --- | --- | --- | --- | --- | --- | --- | --- |
| A17  A81.0   A81.1  F80.3  F84.2   G10   G11.1  G11.3  G12  G20  G23.0  G23.8  G31.8  G31.9  G35  G40.4   G40.5   G60.0   G60.1   G70.2   G70.9   G71.0   G71.1   G71.2   G71.3   G80.0   G80.8   G82.3   G82.4   G82.5   G93.4   G93.6   G93.7 | B20-B24  D56.1  D61.0  D61.9  D76.1  D81  D82.1  D83  D89.1 | C00-C97  D33  D43   D44.4   D48 | E31.0  E34.8  E70.2   E71   E72   E74   E75   E76   E77   E79.1   E83.0   E88.0   E88.1 | E84   J84.1   J96 (excl J96.9)   J98.4 | I21   I27.0   I42   I61.3   I81 | K55.0   K55.9   K72   K74   K76.5   K86.8 | N18 (excl N181, N182, N183)   N19   N25.8 | P10.1   P11.2   P21.0   P28.5   P29.0   P29.3   P35.0   P35.1   P35.8   P37.1  P52.4   P52.5   P52.9   P83.2   P91.2   P91.6   P96.0 | Q00.0  Q01 Q03.1  Q03.9  Q04.0  Q04.2  Q04.3  Q04.4  Q04.6  Q04.9  Q07.0  Q20.0  Q20.3  Q20.4  Q20.6  Q20.8  Q21.3  Q23.2  Q21.8  Q22.0  Q22.1  Q22.4  Q22.5  Q22.6  Q23.0  Q23.4  Q23.9  Q25.4  Q25.6  Q26.2  Q26.4  Q26.8  Q28.2  Q32.1  Q33.6  Q39.6  Q41.0  Q41.9  Q43.7  Q44.2  Q44.5  Q44.7  Q60.1  Q60.6  Q61.4  Q61.9  Q64.2  Q74.3  Q74.8  Q75.0  Q77.2  Q77.3  Q77.4  Q78.0  Q78.5  Q79.2  Q79.3  Q80.4  Q81  Q82.1  Q82.4  Q85.8  Q86.0  Q87.0  Q87.1  Q87.2  Q87.8 Q91  Q92.0  Q92.1  Q92.4  Q92.7  Q92.8  Q93.2  Q93.3  Q93.4  Q93.5  Q93.8  Q95.2 |  | H11.1   H49.8   H35.5   M31.3   M32.1   M89.5   T86.0   T86.2   Z51.5 |

*Supplementary Table 2: Overall numbers and annual prevalence (per 10,000 population) of children (0–19 years) with life-limiting conditions in England by age group for financial years 2003/04–2022/23.*

| Year | Number of patients | Prevalence per 10,000 population | | | | | | | | | | | | |
| --- | --- | --- | --- | --- | --- | --- | --- | --- | --- | --- | --- | --- | --- | --- |
|  |  | **0–19 yrs** | **95% CI** | **<1 yr** | **95% CI** | **1–5 yrs** | **95% CI** | **6–10 yrs** | **95% CI** | **11–15 yrs** | **95% CI** | **16–19 yrs** | **95% CI** |  |
| 2003/04 | 31,298 | 25.3 | 25.0–25.6 | 122.0 | 119.2 - 124.9 | 28.6 | 28.0 - 29.2 | 18.8 | 18.3 - 19.2 | 18.1 | 17.6 - 18.6 | 16.8 | 16.3 - 17.3 |  |
| 2004/05 | 35,860 | 28.9 | 28.6 - 29.2 | 132.8 | 129.9 - 135.7 | 34.9 | 34.3 - 35.6 | 21.0 | 20.5 - 21.5 | 20.8 | 19.5 - 20.5 | 18.9 | 18.4 - 19.4 |  |
| 2005/06 | 41,060 | 33.1 | 32.8 - 33.4 | 139.8 | 136.8 - 142.8 | 41.7 | 40.9 - 42.4 | 24.2 | 23.7 - 24.8 | 23.7 | 22.1 - 23.2 | 22.2 | 21.6 - 22.8 |  |
| 2006/07 | 45,204 | 36.4 | 36.1 - 36.7 | 156.5 | 153.4 - 159.6 | 46.3 | 45.6 - 47.1 | 26.2 | 25.6 - 26.8 | 25.2 | 23.5 - 24.6 | 23.8 | 23.2 - 24.4 |  |
| 2007/08 | 48,995 | 39.2 | 38.9 - 39.6 | 156.5 | 153.5 - 159.5 | 51.0 | 50.2 - 51.8 | 28.5 | 27.9 - 29.2 | 27.3 | 25.5 - 26.7 | 25.7 | 25.1 - 26.3 |  |
| 2008/09 | 53,352 | 42.5 | 42.2 - 42.9 | 175.0 | 171.8 - 178.1 | 53.8 | 53.0 - 54.7 | 30.6 | 30.0 - 31.2 | 29.1 | 27.0 - 28.2 | 27.6 | 26.9 - 28.2 |  |
| 2009/10 | 57,035 | 45.2 | 44.9 - 45.6 | 183.8 | 180.5 - 187.0 | 56.4 | 55.5 - 57.2 | 34.0 | 33.3 - 34.7 | 31.3 | 28.7 - 29.9 | 29.2 | 28.6 - 29.9 |  |
| 2010/11 | 60,707 | 47.9 | 47.5 - 48.3 | 186.8 | 183.5 - 190.0 | 59.0 | 58.2 - 59.8 | 37.3 | 36.6 - 38.0 | 33.6 | 30.8 - 32.0 | 31.0 | 30.4 - 31.7 |  |
| 2011/12 | 61,917 | 48.7 | 48.3 - 49.1 | 170.9 | 167.8 - 174.0 | 60.1 | 59.2 - 60.9 | 40.1 | 39.3 - 40.8 | 32.8 | 32.2 - 33.5 | 31.9 | 31.2 - 32.5 |  |
| 2012/13 | 66,574 | 52.2 | 51.8 - 52.6 | 184.1 | 180.9 - 187.3 | 63.2 | 62.4 - 64.1 | 43.9 | 43.1 - 44.6 | 35.2 | 34.6 - 35.9 | 33.2 | 32.5 - 33.9 |  |
| 2013/14 | 71,060 | 55.5 | 55.1 - 55.9 | 208.8 | 205.4 - 212.2 | 65.7 | 64.8 - 66.5 | 45.7 | 45.0 - 46.5 | 37.4 | 36.7 - 38.1 | 35.3 | 34.6 - 36.1 |  |
| 2014/15 | 74,576 | 58.1 | 57.7 - 58.5 | 218.5 | 215.0 - 222.0 | 67.7 | 66.9 - 68.6 | 48.1 | 47.3 - 48.8 | 40.5 | 39.7 - 41.2 | 37.1 | 36.4 - 37.9 |  |
| 2015/16 | 78,472 | 60.8 | 60.4 - 61.2 | 227.5 | 223.9 - 231.0 | 71.8 | 70.9 - 72.7 | 48.9 | 48.1 - 49.7 | 43.3 | 42.6 - 44.1 | 38.6 | 37.9 - 39.4 |  |
| 2016/17 | 81,095 | 62.5 | 62.1 - 62.9 | 230.5 | 227.0 - 234.1 | 72.7 | 71.8 - 73.6 | 50.4 | 49.6 - 51.1 | 45.8 | 45.1 - 46.6 | 40.2 | 39.4 - 41.0 |  |
| 2017/18 | 83,801 | 64.2 | 63.8 - 64.7 | 227.7 | 224.2 - 231.2 | 74.5 | 73.6 - 75.4 | 51.8 | 51.0 - 52.5 | 48.6 | 47.8 - 49.4 | 42.0 | 41.2 - 42.8 |  |
| 2018/19 | 87,885 | 66.9 | 66.5 - 67.3 | 230.4 | 226.9 - 233.9 | 77.4 | 76.5 - 78.4 | 54.1 | 53.4 - 54.9 | 51.3 | 50.5 - 52.1 | 44.5 | 43.7 - 45.3 |  |
| 2019/20 | 87,898 | 66.4 | 66.0 - 66.8 | 222.7 | 219.2 - 226.2 | 75.2 | 74.3 - 76.2 | 54.8 | 54.0 - 55.6 | 51.3 | 50.6 - 52.1 | 46.0 | 45.2 - 46.8 |  |
| 2020/21 | 67,400 | 50.4 | 50.1 - 50.8 | 190.5 | 187.4 - 193.7 | 52.4 | 51.7 - 53.2 | 40.3 | 39.6 - 40.9 | 39.1 | 38.4 - 39.8 | 37.2 | 36.5 - 38.0 |  |
| 2021/22 | 86,149 | 63.8 | 63.4 - 64.2 | 213.7 | 210.3 - 217.0 | 70.7 | 69.8 - 71.6 | 51.3 | 50.6 - 52.1 | 49.6 | 48.9 - 50.4 | 48.3 | 47.4 - 49.2 |  |
| 2022/23 | 88,722 | 65.0 | 64.6 - 65.4 | 209.1 | 205.8 - 212.4 | 70.2 | 69.3 - 71.1 | 55.2 | 54.4 - 56.0 | 51.2 | 50.4 - 51.9 | 49.5 | 48.6 - 50.3 |  |

Supplementary Table 3: Annual prevalence (per 10,000 population) of children (0–19 years) with life-limiting conditions in England by diagnostic group for financial years 2003/04–2022/23.  A child or young person with multiple diagnoses could have a life-limiting condition in more than one category.

| Year | Neurology | Haemotology | Oncology | Metabolic | Respiratory | Circulatory | Gastrointestinal | Genitourinary | Perinatal | Congenital | Other |
| --- | --- | --- | --- | --- | --- | --- | --- | --- | --- | --- | --- |
| **2003/04** | 3.9 | 2.7 | 4.6 | 1.2 | 3.4 | 0.8 | 0.4 | 1.2 | 1.5 | 9.0 | 0.4 |
| **2004/05** | 4.5 | 3.6 | 5.1 | 1.4 | 3.9 | 1.1 | 0.7 | 1.5 | 2.1 | 10.4 | 0.4 |
| **2005/06** | 5.4 | 4.3 | 5.6 | 1.6 | 4.4 | 1.4 | 0.8 | 1.8 | 2.5 | 12.2 | 0.5 |
| **2006/07** | 6.0 | 4.8 | 5.7 | 1.8 | 4.7 | 1.6 | 1.0 | 2.0 | 3.5 | 13.8 | 0.5 |
| **2007/08** | 6.5 | 5.2 | 6.0 | 2.0 | 5.2 | 1.8 | 1.2 | 2.1 | 3.9 | 15.1 | 0.6 |
| **2008/09** | 7.0 | 5.4 | 6.2 | 2.1 | 6.1 | 1.9 | 1.4 | 2.2 | 4.9 | 16.2 | 0.7 |
| **2009/10** | 7.5 | 5.7 | 6.3 | 2.4 | 6.2 | 2.3 | 1.5 | 2.4 | 5.6 | 17.7 | 0.8 |
| **2010/11** | 8.0 | 6.3 | 6.5 | 2.5 | 6.5 | 2.4 | 1.8 | 2.5 | 6.1 | 19.1 | 0.9 |
| **2011/12** | 8.3 | 6.5 | 6.5 | 2.7 | 7.1 | 2.6 | 1.9 | 2.5 | 5.9 | 19.8 | 1.0 |
| **2012/13** | 9.0 | 7.1 | 6.8 | 2.9 | 7.7 | 2.4 | 2.0 | 2.7 | 7.1 | 21.1 | 1.0 |
| **2013/14** | 9.5 | 7.3 | 6.9 | 3.1 | 8.0 | 2.3 | 2.1 | 2.8 | 8.5 | 23.0 | 1.2 |
| **2014/15** | 10.0 | 7.7 | 7.2 | 3.3 | 8.7 | 2.4 | 2.3 | 2.9 | 9.2 | 23.9 | 1.3 |
| **2015/16** | 10.4 | 8.1 | 7.4 | 3.5 | 9.1 | 2.3 | 2.5 | 2.9 | 10.4 | 25.0 | 1.4 |
| **2016/17** | 10.6 | 8.3 | 7.5 | 3.7 | 9.7 | 2.3 | 2.6 | 2.9 | 11.0 | 25.9 | 1.6 |
| **2017/18** | 10.8 | 8.7 | 7.6 | 3.9 | 10.2 | 2.3 | 2.6 | 2.9 | 11.3 | 27.0 | 1.7 |
| **2018/19** | 11.1 | 9.0 | 7.8 | 4.1 | 10.9 | 2.4 | 2.9 | 3.1 | 12.2 | 28.2 | 1.9 |
| **2019/20** | 11.2 | 9.0 | 7.7 | 4.2 | 10.9 | 2.3 | 2.9 | 3.0 | 12.3 | 28.2 | 1.9 |
| **2020/21** | 8.5 | 7.7 | 6.8 | 3.3 | 7.6 | 1.7 | 2.3 | 2.3 | 9.5 | 21.5 | 1.7 |
| **2021/22** | 10.7 | 9.1 | 7.8 | 4.2 | 10.3 | 2.2 | 2.8 | 2.8 | 12.4 | 26.9 | 2.2 |
| **2022/23** | 10.7 | 9.5 | 7.8 | 4.4 | 10.9 | 2.2 | 2.8 | 2.8 | 12.3 | 27.6 | 2.2 |
| **% increase 2003/4-2022/23** | 277% | 350% | 168% | 369% | 326% | 256% | 647% | 232% | 844% | 305% | 605% |

Supplementary Table 4 Composite complexity score by age group for 2022/23 data.

|  | **0** | **1** | **2** | **3** | **4** | **5** | **Total** |
| --- | --- | --- | --- | --- | --- | --- | --- |
| <1 | 6657 (44.6%) | 3542 (23.7%) | 2462 (16.5%) | 1540 (10.3%) | 606 (4.1%) | 107 (0.7%) | 14,914 (100%) |
| 1–5 | 5114 (20.9%) | 7527 (30.7%) | 6235 (25.4%) | 3789 (15.5%) | 1532 (6.3%) | 321 (1.3%) | 24,518 (100%) |
| 6–10 | 2991 (15.8%) | 6439 (34.1%) | 5297 (28.0%) | 3012 (16.0%) | 968 (5.1%) | 180 (1.0%) | 18,887 (100%) |
| 11–15 | 2369 (13.4%) | 5913 (33.5%) | 5286 (30.0%) | 3016 (17.1%) | 918 (5.2%) | 121 (0.7%) | 17,623 (100%) |
| 16–19 | 1683 (13.2%) | 4076 (31.9%) | 3962 (31.0%) | 2064 (16.1%) | 801 (6.3%) | 194 (1.5%) | 12,780 (100%) |
| 0–19 | 18814 (21.2%) | 27497 (31.0%) | 23242 (26.2%) | 13421 (15.1%) | 4825 (5.4%) | 923 (1.0%) | 88,722 (100%) |
